# Transcriptome profiling reveals distinctive traits of retinol metabolism and neonatal parallels in the MRL/MpJ mouse

**DOI:** 10.1186/s12864-015-2075-2

**Published:** 2015-11-14

**Authors:** Justyna Podolak-Popinigis, Bartosz Górnikiewicz, Anna Ronowicz, Paweł Sachadyn

**Affiliations:** Department of Molecular Biotechnology and Microbiology, Gdańsk University of Technology, Gdańsk, Poland; Department of Biology and Pharmaceutical Botany, Medical University of Gdańsk, Gdańsk, Poland

**Keywords:** Regeneration, Retinol, MRL/MpJ mouse, Transcriptome profiling, Gene expression microarray, Neonatal parallels, Peroxisome proliferator-activated receptor

## Abstract

**Background:**

The MRL/MpJ mouse is a laboratory inbred strain known for regenerative abilities which are manifested by scarless closure of ear pinna punch holes. Enhanced healing responses have been reported in other organs. A remarkable feature of the strain is that the adult MRL/MpJ mouse retains several embryonic biochemical characteristics, including increased expression of stem cell markers.

**Results:**

We explored the transcriptome of the MRL/MpJ mouse in the heart, liver, spleen, bone marrow and ears. We used two reference strains, thus increasing the chances to discover the genes responsible for the exceptional properties of the regenerative strain. We revealed several distinctive characteristics of gene expression patterns in the MRL/MpJ mouse, including the repression of immune response genes, the up-regulation of those associated with retinol metabolism and PPAR signalling, as well as differences in expression of the genes engaged in wounding response. Another crucial finding is that the gene expression patterns in the adult MRL/MpJ mouse and murine neonates share a number of parallels, which are also related to immune and wounding response, PPAR pathway, and retinol metabolism.

**Conclusions:**

Our results indicate the significance of retinol signalling and neonatal transcriptomic relics as the distinguishing features of the MRL/MpJ mouse. The possibility that retinoids could act as key regulatory molecules in this regeneration model brings important implications for regenerative medicine.

**Electronic supplementary material:**

The online version of this article (doi:10.1186/s12864-015-2075-2) contains supplementary material, which is available to authorized users.

## Background

The MRL/MpJ inbred strain of mouse is one of few exceptions among adult mammals which shows enhanced ability to regenerate different organs and tissues. An initial finding that the MRL/MpJ mouse is able to close through-and-through holes made in ear pinnae within 30 days after injury without scarring, was followed by the observations that the process involved rapid re-epithelisation, blood vessels and hair follicles recovery, intensive growth of peripheral nerves, and cartilage regeneration. The repair is mediated by the formation of blastema-like structure [1, 2]. Further studies reported the ability of the MRL/MpJ mouse to regenerate spinal cord after dorsal hemisection [3], accelerated digit tip re-growth after amputation [4, 5], more permissive environment for retina regeneration after *in vitro* retinal explanation [6], enhanced regeneration of retinal pigment epithelium after ablation with sodium iodate [7], and accelerated healing of alkali-burned cornea [8]. Also, scarless heart healing has been reported in a few articles [9–12], but other studies either have not confirmed this result or reported only limited heart healing in the MRL/MpJ mouse [13–17].

The regenerative capacity observed in different tissues of the MRL/MpJ mouse has been investigated in several independent laboratories in order to examine and explain the mechanisms of this phenomenon. Previous studies of the MRL/MpJ mouse ear hole closure, cardiac cryoinjuries, thermal skin injuries and digit tip amputation have shown greater collagen synthesis along with enhanced matrix metalloproteinase activity in the wound area [1, 5, 9, 18, 19]. Increased expression of proteases leads to basement membrane breakdown, thus preventing scarring and enabling the formation of blastema-like structure, which is probably the critical step in the regenerative process [18]. Genetic linkage analyses indicate that the “heal” trait is multigenic [20].

In addition to the healing capacity, the MRL/MpJ mouse has been found to display a number of distinctive characteristics such as increased size, autoimmunity, the existence of mitochondrial heteroplasmy [21], natural resistance to high fat diet-induced hyperglycaemia [22], and uncommon cell cycle profile [23]. Another exceptional feature of the MRL/MpJ mouse is retaining of selected embryonic features in adults including the expression of pluripotency markers genes such as *Nanog, Islet-1* and *Sox2* [24]. Genome-wide microarray profiling showed that DNA methylation levels in the promoter regions of a number of genes responsible for embryonic development were decreased in the MRL/MpJ versus the reference C57BL/6 J strain [25].

Several transcriptomic studies for the tissues collected from injured heart, digits and ear have been conducted in order to identify the genes differentially expressed in the MRL/MpJ mouse in comparison to the reference strains which do not display enhanced regenerative capability [4, 10, 11, 26]. Naseem *et al*. reported enhanced levels of genes connected with vasculogenesis and those encoding cardiac structural proteins, signalling, growth factors and cell cycle regulators in the injured hearts of the MRL/MpJ mouse as compared to the C57BL/6J controls [11]. Another study addressing the transcriptomic profiles in the heart after infarction [10] showed that the genes connected with immune response and apoptosis were down-regulated in the MRL/MpJ mouse in comparison to the C57BL/6J strain during healing, whereas those related to reparative processes were up-regulated [10]. The comparison of transcriptomic profiles during digit tip re-growth in the MRL/MpJ and the CBA and C57BL/6J control strains revealed a group of 75 differentially expressed genes including the *Lrp6,* a WNT co-receptor which functions in limb morphogenesis [4]. Masinde *et al*. identified 36 genes differentially expressed in the MRL/MpJ mouse during ear wound healing relative to the C57BL/6J control. Six among those genes were known to have a documented role in wound healing [26]. In another study, Li *et al*. showed that genes associated with inflammatory response are down-regulated during ear hole closure in the MRL/MpJ mouse, whereas increased proliferation profile is observed in comparison to the C57BL/6J mouse [27]. The transcriptomic analyses revealed several sets of genes differentially expressed in the MRL/MpJ mouse in response to injury but they do not expose a consistent transcriptomic signature which may distinguish the MRL/MpJ mouse from other murine strains. Nevertheless, all these studies conclude that the healing process in the MRL/MpJ mouse is connected with the repression of genes responsible for inflammation and induction of those which mediate proliferation.

With a view to gaining a better insight into the unusual properties of the MRL/MpJ mouse, we designed a comprehensive comparative analysis of genome-wide expression profiles in ears, heart, spleen, liver and bone marrow of the MRL/MpJ mouse and two control strains, the C57BL/6J and BALB/c. Transcriptomic studies in the mouse are based usually on contrasting the examined transcriptome to a single reference. Such approach is useful with regard to compare a knock-out mouse with the wild type strain, but in the case of the MRL/MpJ mouse there is no control strain developed on the same genetic background but deprived of enhanced healing abilities. This is why, in order to identify a distinguishing transcriptomic signature of the MRL/MpJ mouse we employed two different reference strains. Such approach not only shows the transcriptomic results in a broader context but it also restricts the analysis to the most essential differences.

As the result, we revealed the characteristics distinguishing the transcription patterns of the MRL/MpJ mouse such as the up-regulation of genes engaged in PPAR signalling and retinol metabolism, the repression of immune response genes and a number of parallels between the MRL/MpJ adults and neonatal mice.

## Results and discussion

We performed genome-wide gene expression profiling in the ear, spleen, liver, heart and bone marrow of 8-week-old females of the MRL/MpJ and two reference strains of C57BL/6J and BALB/c by using a high-density microarray platform (NimbleGen mouse gene expression 12x135K array) which interrogates 44,170 transcripts corresponding to over 24,200 genes. With regard to obtaining transcriptomic profiles representative of the strains and tissues, each organ was powdered prior to RNA extraction and RNA pools from three animals of each strain were used for the microarray experiments. Additionally, RNA-Seq was used as a reference method to obtain the transcriptomic profiles representing single hearts of each of the three examined murine strains.

### Selection of differentially expressed genes from microarray data

**Fig. 1:**
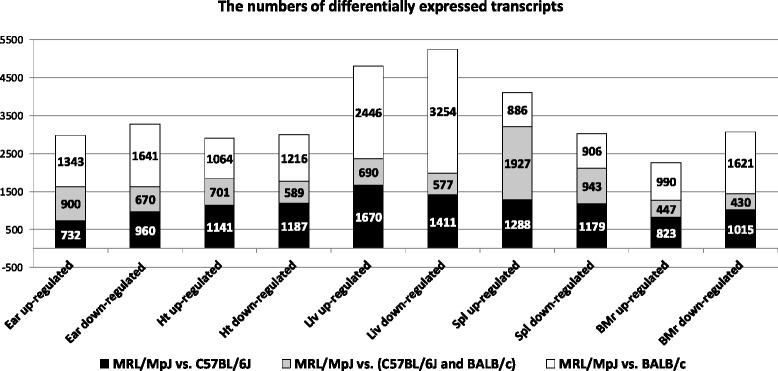
The numbers of transcripts differentially expressed in the tissues of the MRL/MpJ mouse. The numbers of transcripts exhibiting at least a two-fold difference in expression level in the MRL/MpJ mouse relative to the reference strains C57BL/6J and BALB/c. The tissues were designated as following: Ht- Heart, Liv - Liver, Spl - Spleen, BMr -bone marrow

We focused the analysis on the transcripts showing at least a two-fold difference in expression between the MRL/MpJ mouse and the two control strains in a given tissue. Such transcripts will be further referred to as the differentially expressed. Thousands of transcripts were found to show at least a two-fold difference in expression when compared within pairs of strains, while hundreds of those when the MRL/MpJ was contrasted against both control strains (Fig. 1). The numbers of the up- and down-regulated transcripts were different but neither was predominant. The number of the transcripts differentially expressed in the spleen of the MRL/MpJ mouse remarkably exceeds those in other tissues. The overall inter-strain similarities between the gene expression profiles were visualized by scatter plots (Additional file 1: Figure S1). A hierarchical clustering analysis was performed in order to visualize the inter-strain differences for the genes which show at least a 2-fold difference in expression between the MRL/MpJ and the two control strains (Additional file 1: Figure S2).

### The transcripts differentially expressed in all examined tissues of the MRL/MpJ mouse

We expect that the transcripts revealing differential expression in all analysed tissues of the MRL/MpJ mouse could be of particular importance, as such differences may reflect the most distinguishing traits of the MRL/MpJ mouse. By microarray analysis, we identified 28 transcripts differentially expressed with at least a 2-fold difference in expression between the MRL/MpJ mouse and the two control strains in all examined tissues. Of these 28 transcripts, 4 were up- and 23 down-regulated (Additional file 1: Table S1)*.* Among the down-regulated ones, two genes attract a particular attention owing to their functions: *Cradd* (component of death pathway), a gene that plays a role in proteolysis and *Nsun3,* encoding an RNA methyltransferase. A long non-coding RNA transcript of unknown function designated as BC044745 is expressed one to two orders of magnitude higher in the MRL/MpJ as compared to the control strains, which deserves further attention.

It is worth to add that though the NimbleGen platform, we applied, covers the majority of reference transcripts, the transcript identifiers used for this microarray indicate one of targeted transcripts, and it is rarely the reference one. The search with probe sequences is necessary in order to find out all targeted transcripts. This is why we decided to refer to official gene names (all transcript identifiers mentioned in the article are listed in Additional file 2).

### Gene ontology analyses

**Fig. 2:**
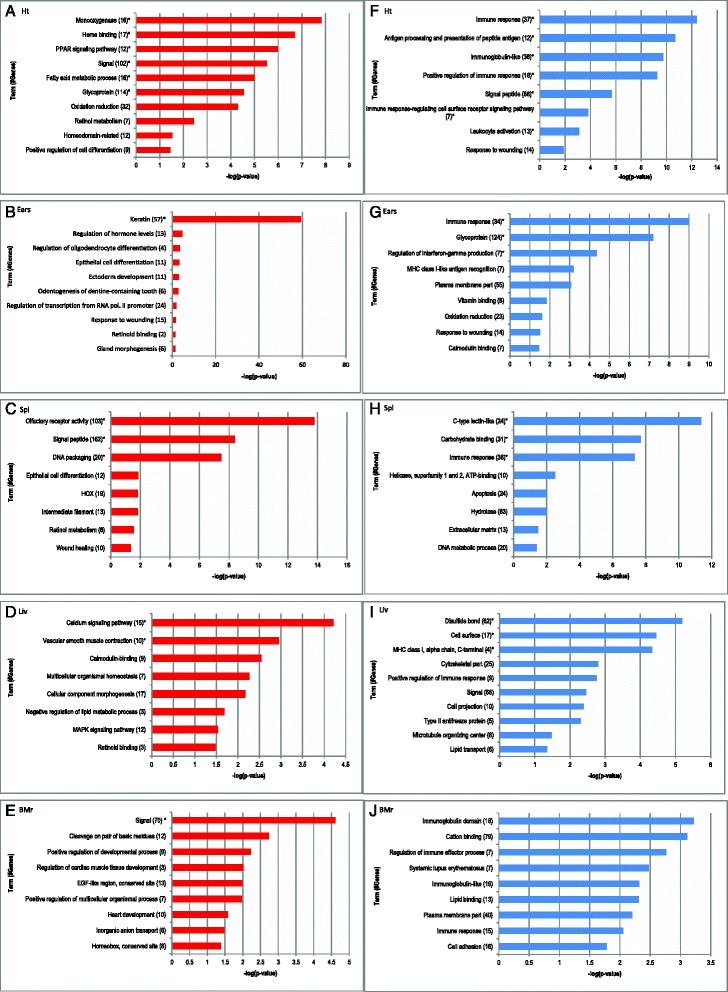
Remarkable pathways associated with the genes differentially expressed in the tissues of the MRL/MpJ mouse. The functional terms associated with the genes enriched among the transcripts up- (red) and down-regulated (blue) in the MRL/MpJ mouse in comparison with the C57BL/6J and BALB/c reference strains. **a, f** heart, **b, g** ears, **c, h** spleen, **d, i** liver, **e, j** bone marrow. The statistical significance of enrichment was determined with Fisher’s exact test and all presented terms have *p*-value < 0.05. The histograms represent the negative decimal logarithm of *p*-value. The terms significant after Benjamini correction are marked with a star "*"

Gene ontology analyses were performed for the gene sets which were found to exhibit at least a two-fold difference in expression between the MRL/MpJ mouse and both control strains. By using the Database for Annotation, Visualization and Integrated Discovery (DAVID v6.7 [28]), we carried out an ontology analysis based on molecular and cellular functions of the genes differentially expressed in the MRL/MpJ mouse. The presented pathways were selected based on the high enrichment score and potential significance in wound healing and regeneration. All ontological terms listed in Fig. 2 and Tables 1, 2, 3, 4, 5 and 6 were statistically significant as confirmed by Fisher’s exact test (*p*-value < 0.05).

**Table 1:** Enrichment of keratin genes among the transcripts up-regulated in the ear of the MRL/MpJ mouse

Up-regulated keratin genes enriched in the ear of the MRL/MpJ mouse	
SP_PIR_KEYWORDS Keratin^**a**^	
*1110025L11Rik, 5430421N21Rik, AY026312, Gm10228, Gm10229, Gm11554, Gm11563, Gm11564, Gm11567, Gm11595, Gm11937, Gm11938, Gm2692, Gm7288, Gm7574, Krt18, Krt25, Krt26, Krt28, Krt32, Krt33a, Krt33b, Krt34, Krt35, Krt36, Krt40, Krt71, Krt72, Krt73, Krt81, Krt82, Krt84, Krt85, Krtap10-4, Krtap12-1, Krtap1-3, Krtap13-1, Krtap14, Krtap15, Krtap16-1, Krtap16-10, Krtap16-4, Krtap16-5, Krtap16-8, Krtap17-1, Krtap2-4, Krtap26-1, Krtap3-1, Krtap3-2, Krtap3-3, Krtap4-16, Krtap4-2, Krtap4-6, Krtap4-7, Krtap4-8, Krtap5-1, Krtap5-4, Krtap5-5, Krtap6-1, Krtap7-1, Krtap8-1, Krtap9-1, Krtap9-3, Krtap9-5*	

The up-regulated genes encoding keratin proteins in the ear of the MRL/MpJ mouse in comparison to the control C57BL/6 J and BALB/c strains. ^a^The gene enrichment was statistically significant as confirmed by Fisher’s exact test with *p*-value of 1.87E-57 after Benjamini correction.

**Table 2:**
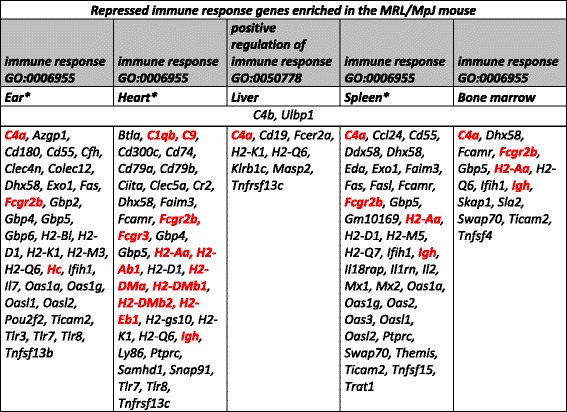
Repression of immune response genes in the MRL/MpJ mouse

The genes encoding immune response factors repressed in the tissues of the MRL/MpJ mouse in comparison to the control C57BL/6J and BALB/c strains. The terms significant after Benjamini correction are marked with a star “*”. The genes associated with systemic lupus erythematosus are shown in red font

**Table 3:**
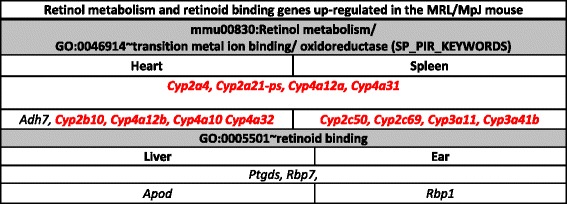
Retinol and retinoid binding genes up-regulated in the tissues of the MRL/MpJ mouse

The genes up-regulated in the MRL/MpJ mouse tissues in comparison to the control C57BL/6J and BALB/c strains clustered by gene ontology analysis into retinol metabolism and retinoid binding pathways. The genes encoding proteins belonging to cytochrome P450 are shown in red font

**Table 4:** Homeobox genes up-regulated in the tissues of the MRL/MpJ mouse

Homeobox genes up-regulated in the MRL/MpJ mouse		
SM00389:HOX	IPR012287:Homeodomain-related	IPR017970:Homeobox, conserved site
Spleen	Heart	Bone marrow
*Meis2, Rhox2e*		*Crx, Gm7148, Gsc2, Hoxd10, Irx1, Irx4, Nkx2-6, Pbx4*
*4933403O03Rik, Dux, Crxos1, Gm3977, Gm3984, Gm3980, Gm3987, Gm3994, Gm3981, Gm4023, Gm4745, Hoxc6, Isl1, Lbx1, Obox2, Obox3, Gm8053, Gm5889, Gm8040, Obox5, Otx2, Rhox2b, Rhox2c, Rhox2a, Rhox2f, Rhox2g, Rhox3g, Rhox3h, Rhox4a, Rhox4d*	*Arx, Cyp4a10, Cyp4a32, Cyp4a31, Gm8743, Gsc2, Hoxc5, Nanog, Pax6, Pbx1, Pou6f2, Sebox*	

The genes up-regulated in the heart, spleen and bone marrow of the MRL/MpJ mouse in comparison to the control strains clustered by gene ontology analysis into homeobox terms

**Table 5:**
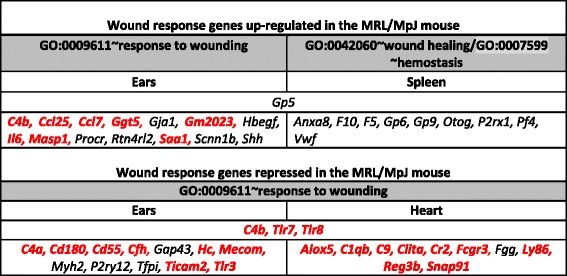
Enrichment of wounding response genes among the transcripts differentially regulated in the MRL/MpJ mouse

A number of wound response and wound healing genes are either up- or down-regulated in the noninjured tissues of the MRL/MpJ mouse in comparison to the control C57BL/6J and BALB/c strains. The genes associated with inflammatory response are shown in red font

**Table 6:**
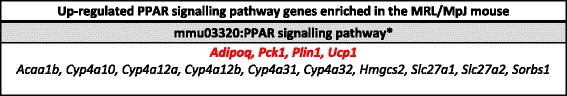
The genes of PPAR signalling pathway up-regulated in the heart of the MRL/MpJ mouse

The genes up-regulated in the MRL/MpJ mouse heart tissue in comparison to the control C57BL/6J and BALB/c strains clustered by gene ontology analysis into PPAR signalling pathway. The terms significant after Benjamini correction are marked with a star “*”. The differential expression of key markers of PPAR signalling shown in red was confirmed by RNA-Seq results

### Up-regulation of keratin genes in the ear tissue of the MRL/MpJ mouse

An extreme enrichment in keratin genes (Table 1) among those up-regulated in the ear of the MRL/MpJ mouse is one of the most conspicuous findings in this study. The statistical significance of this enrichment exceeds by far other results of functional annotation (Fig. 2b). Enhanced expression of keratin genes have been already reported in the MRL/MpJ as compared to the C57BL/6J mouse in the digit [29]. The properties of keratins applied in wound dressings may implicate that increased keratin levels in the MRL/MpJ mouse promote regeneration. On the other hand, it should be underlined that dorsal skin lesions in the MRL/MpJ mouse have been reported to heal with scarring [30].

### Repression of immune response genes

Among the genes down-regulated in the MRL/MpJ mouse we distinguished the gene clusters connected with inflammation and immune response which were found in the five examined tissues (Fig. 2f, g, h, i, j; Table 2). As the MRL/MpJ mouse is known to be prone to autoimmune diseases [31], the differences in the expression of genes involved in immune response seem to correspond to this feature of the strain. The susceptibility to systemic lupus erythematosus is a known trait of the MRL/MpJ strain [32] and it could be connected with the repression of several genes associated with this disease in the spleen and bone marrow of the MRL/MpJ mouse (Table 2). Further, immune response and inflammation have been known to play an essential role in wound healing and there is growing knowledge on immune system involvement in regeneration processes [8, 33, 34].

### Retinol metabolism and retinoid binding pathway genes

We have observed the up-regulation of retinol metabolism and retinoid binding protein genes in spleen, heart, ears and liver tissues of the MRL/MpJ mouse (Fig. 2a, b, c, d). Among these genes, those found in the heart and spleen belong to the cytochrome P450 family (Table 3) and they might be involved in the degradation of excess retinoic acid, while those identified in ear and liver tissues are highly expressed genes which are responsible for retinol binding (*Rbp7* [35], *Rbp1* [36], and *Ptgds* [35]). Prostaglandin D2 synthase (*Ptgds*) binds all-*trans*- and 9-*cis*-retinoic acid and all-*trans*- and 13-*cis*-retinal with the same affinity as other retinoid transporters [37]. Cellular retinol-binding protein 1 (*Rbp1*, also known as CRBP1) plays an important role in the conversion of retinol to retinyl esters for storage in liver and to facilitate retinol oxidation to retinaldehyde by retinaldehyde dehydrogenases [38, 39].

The genes of retinoid binding proteins are up-regulated in all the examined tissues of the MRL/MpJ mouse. While *Rbp1* is up-regulated in the spleen and bone marrow of the MRL/MpJ mouse, an enhanced expression of *Rbp7* transcript is observed in the heart, liver and ears. Assuming that increased levels of retinoid binding proteins are associated with more effective retinol internalization, the tissues of the MRL/MpJ mouse could display an enhanced sensitivity to retinoid stimulation.

Another important enzyme, which regulates retinoic acid metabolism, is alcohol dehydrogenase (Adh7). It catalyses the conversion of all-*trans*-retinol to all-*trans*-retinal and it mainly plays an important role in postnatal growth and development [40]. In the liver, we also can observe an elevated level of *Apod* (apolipoprotein D) transcript, the expression of which is induced by retinoic acid and which has been reported to participate in tumorigenesis [41]. It was shown that the genes of *Cyp4* family, which belong to the cytochrome P450 group, were involved in the metabolism of specific groups of biologically active compounds as steroid hormones, bile acids and vitamins A and D [42]. Also the family of *Cyp3a* genes, which are expressed from embryonic day 11 [43], might be responsible for all-*trans* -retinoic acid metabolism [44].

**Fig. 3:**
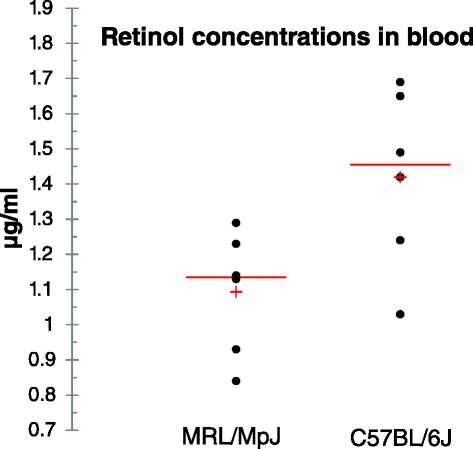
The concentrations of blood retinol in the MRL/MpJ mouse relative to the C57BL/6J reference. The mean retinol concentrations determined for the C57BL/6J and MRL/MpJ mouse, were 1.42 (*n* = 6) and 1.09 μg/ml (*n* = 6), respectively. The accuracy of the measurement method was estimated as 0.06 μg/ml. The mean difference in concentrations was statistically significant as determined by homoscedastic two-tailed Student’s *t*-test (*p*-value = 0.026)

Retinoic acid regulates transcription as a ligand for nuclear receptors that bind DNA [38]. One of its key functions is to control cellular differentiation. The role of retinoic acid signalling has been well established in the model of limb regeneration in amphibians [45, 46] and fin regrowth in adult zebrafish [47]. In mammals, retinoic acid signalling has been extensively studied in embryonic development, organogenesis [36, 38], and wound healing [48]. The role of retinoids has also been examined in regeneration of deer antlers [49]. Our transcriptomic data indicate distinctive characteristics of retinol metabolism in another model of mammalian regeneration, the MRL/MpJ mouse. The expression patterns of genes responsible for retinoid binding and conversion suggest accelerated metabolism of retinoids in the MRL/MpJ mouse. Indeed, we observed statistically significant lower levels of blood retinol concentrations in the MRL/MpJ relative to the C57BL/6J mouse (Fig. 3).

### Homeobox clusters

The activity of homeobox genes is crucial in the course of development since they encode the positional information expressed during primary and secondary axis formation. Homebox genes have been also reported to be active during development [50] and regeneration [51, 52]. We found that several hox genes were up-regulated in the heart, spleen and bone marrow of the MRL/MpJ mouse (Fig. 2a, c, e; Table 4). We can distinguish the *Meis2* gene which together with *Meis1* are activated by retinoic acid during PD (proximal-distal) axis formation [53, 54]. Also, Meis2 is critical for the proper heart tube formation and cardiac looping during development [55], as well as during limb regeneration in axolotl [56]. *Meis1* and *Pbx1* play a role as homeobox cofactors [57]. Moreover, in the heart of the MRL/MpJ strain, we can observe an up-regulation of the *Nanog* gene, one of key pluripotency markers.

The enhanced expression of homeobox genes in the MRL/MpJ mouse is in agreement with previous observations which indicated that the MRL/MpJ mouse retained a selection of embryonic features. Noteworthy, the expression of selected homeobox genes has been reported to show a much greater increase in the MRL/MpJ mouse after injury than in the control strain [24].

### Differential expression of genes associated with response to wounding in noninjured tissues of the MRL/MpJ mouse

A remarkable result to emerge from the gene ontology analysis for both the up- and down-regulated transcripts in the ear of the MRL/MpJ mouse is the enrichment of genes associated with response to wounding (Fig. 2b, g; Table 5). The genes associated with this term are enriched among the transcripts down-regulated in the heart (Fig. 2f, Table 5) of the MRL/MpJ, including three genes (*C4b, Tlr7, Tlr8*) shared with those which are also repressed in the ear (Table 5). A prevailing part of the listed genes associated with wound healing are involved in inflammatory response, including the interleukin 6 gene (*Il6*) known as a key factor during skin wound healing [58] and ear wound healing [59]. It is worth noting that improved wound repair could be connected with deficiencies of genes involved in wound healing [60–63].

“Wound healing”, the term relative to “wounding response”, was found to be associated with the genes up-regulated in the spleen of the MRL/MpJ mouse (Fig. 2c, Table 5). All genes belonging to this set are responsible for blood coagulation in the haemostasis phase of wound response.

It is worth to note an enhanced expression of the *Shh* gene encoding a well-known morphogen in the ear tissue of the MRL/MpJ mouse. The gene has been reported to be crucial during limb regeneration after amputation in newts [64] and *Xenopus laevis* tadpole [65].

### PPAR signalling pathway genes activated in the heart of the MRL/MpJ mouse

We observed an overrepresentation of the genes connected with peroxisome proliferator-activated receptor (PPAR) signalling pathway among the transcripts up-regulated in the heart of the MRL/MpJ mouse (Fig. 2a). The finding is confirmed by the results obtained from both microarray and RNA-Seq analyses. PPARs participate in the combustion and storage of lipids and adipogenesis and they are known to act in some regenerative processes such as PPARs regulated satellite cell proliferation and skeletal muscle regeneration [66], and epidermal wound repair [67].

Two key markers of PPAR signalling induction were greatly up-regulated in the MRL/MpJ mouse: adiponectin and uncoupling protein 1. Adiponectin (encoded by the *Adipoq* gene) (Table 6), is one of the key adipose-specific secretary factors. It was shown that the *Adipoq* gene is expressed during mouse embryogenesis from embryonic day 16.5 and its concentration grows at birth. Adiponectin is expressed in brown adipocyte tissue and surrounding immature tissue in the foetus. It was speculated that adiponectin may have a role in endocrine function in embryos [68]. Moreover, adiponectin might be an effective mediator in the regulation of cutaneous wound healing [69]. Another important marker of adipogenesis is uncoupling 1 protein (UCP1). *UCP1* is expressed in brown fat and it is mainly responsible for non-shivering thermogenesis [70]. Nevertheless, it may also play a function in heart protection from a specific type of damage. It was shown that the H9c2 cells, derived from embryonic heart tissue, transfected with *UCP1* exhibited higher cardioblast survival and limited ROS formation following hypoxia [71]. Therefore UCP1 may play a role in heart protection from severe hypothermia [70].

### Neonatal parallels of gene expression profiles in the MRL/MpJ mouse

Embryos and neonates are known for enhanced regenerative potential, similarly as the adult MRL/MpJ mouse is. With regard to investigating this resemblance, we compared the transcriptomic profiles in the adult hearts of the MRL/MpJ and of the control strains with those of day 1 and 7 newborns in addition to those of day 15–19 embryos of the C57BL/6J mouse (The heart gene expression profiles are deposited in Gene Expression Omnibus Database under accession number GSE68524 (http://www.ncbi.nlm.nih.gov/geo/query/acc.cgi?token=klgvsskqpvsplgn&acc=GSE68524).

In order to track for the neonatal and embryonic paralleles in the adults of the MRL/MpJ mouse, we focused on the transcripts which were differentially regulated in newborns, embryos and the MRL/MpJ adults relative to the adults of the control strains. Analogically, as in other analyses, we applied a cut-off of two-fold difference in expression.

**Fig. 4:**
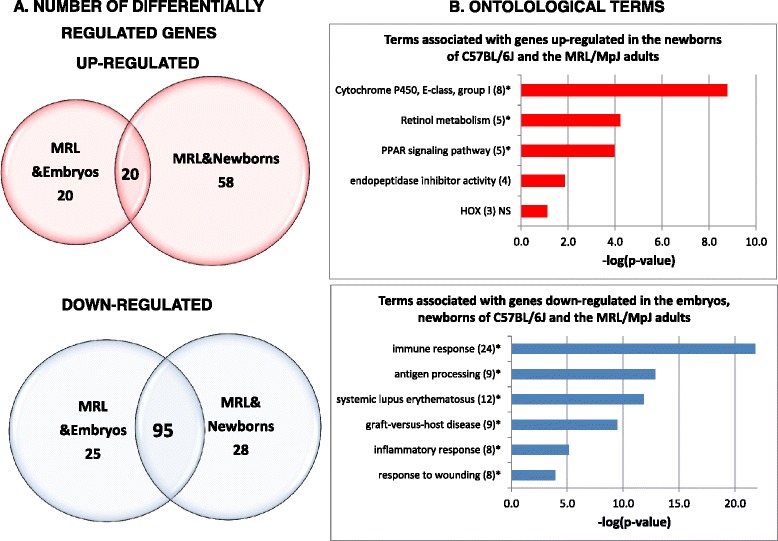
Neonatal transcriptomic parallels in the MRL/MpJ adults. **a** The numbers of down- and up-regulated genes in the hearts of MRL/MpJ adults, embryos and neonates of C57BL/6 J relative to the adults of C57BL/6J. **b** Remarkable pathways associated with the transcription patterns shared by embryos, neonates and MRL/MpJ adults. The terms significant after Benjamini correction are marked with a star "*"

The group of down-regulated transcripts shared by the adults of MRL/MpJ with the neonates largely overlapped with that shared by the adults of MRL/MpJ with embryos. Analogical groups of up-regulated transcripts were mostly not overlapping (Fig. 4a, Additional file 2). Several homeobox genes were up-regulated in the MRL/MpJ adults, embryos and neonates. However, there is a group of transcripts up-regulated in the MRL/MpJ adults and the newborns, but not in the embryos. The group is enriched with the genes associated with retinol metabolism and PPAR signalling in addition to those encoding peptidase inhibitors (Fig. 4b, Table 7). We may conclude that the MRL/MpJ mouse betray both embryonic and neonatal features, but these are the latter ones which seem more pronounced.

**Table 7:**
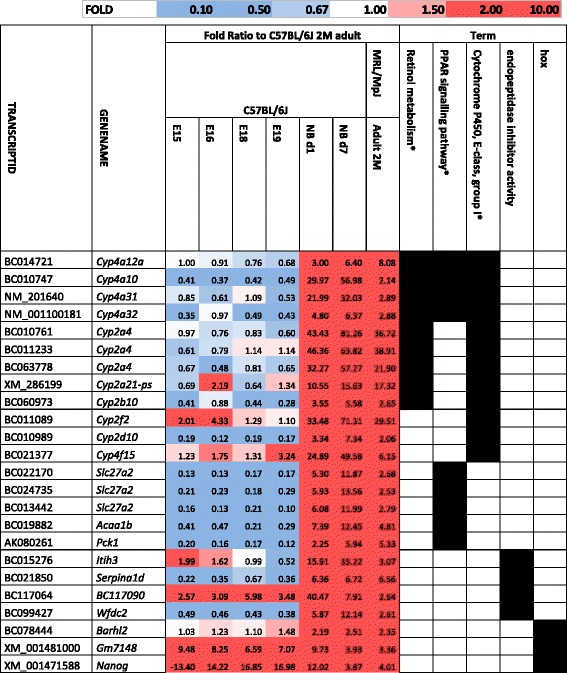
Neonatal and embryonic parallels characteristic of the transcription patterns of genes up-regulated in the heart of the MRL/MpJ mouse

The gene expression profiles in the hearts of adult MRL/MpJ mouse and the control strains were contrasted with those of the embryos (E15-E19) and neonates (d1, d7). The comparison exposed a gene expression pattern characteristic of significant enrichment with the genes of immune and wounding response, retinol metabolism and PPAR signalling

The terms significant after Benjamini correction are marked with a star "*"

The down-regulated transcripts were greatly enriched with immune response genes including those associated with systemic lupus erythematosus, antigen processing, Graft-versus host disease, and response to wounding (Fig. 4b, Table 8). All these genes associated with response to wounding are also engaged in inflammatory response. The transcriptional repression of a number of immune response genes both in the adults MRL/MpJ mouse and the embryos and neonates of the control strain entails the question as to whether it is the immature immune system which contributes to improved healing in the MRL/MpJ mouse as it does in embryos and neonates.

**Table 8:**
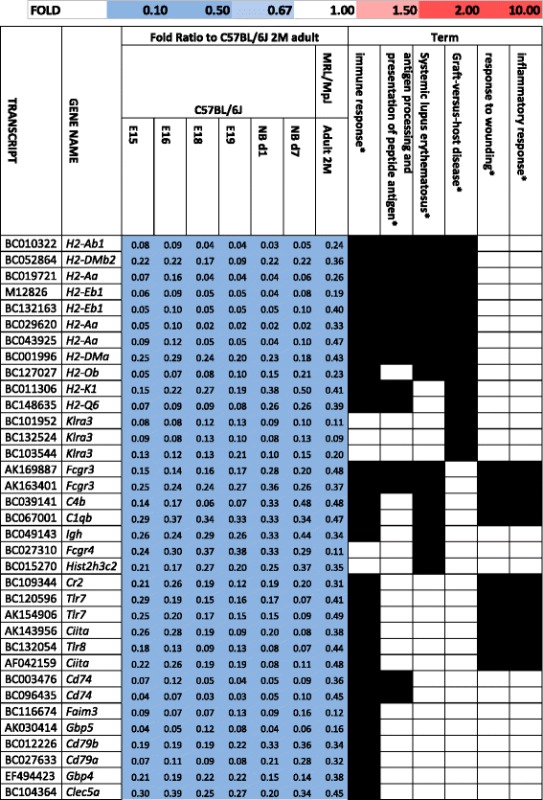
Neonatal and embryonic parallels characteristic of the transcription patterns of genes down-regulated in the heart of MRL/MpJ mouse

The gene expression profiles in the hearts of adult MRL/MpJ mouse and the control strains were contrasted with those of the embryos (E15–E19) and neonates (d1, d7)

The terms significant after Benjamini correction are marked with a star "*"

Regarding to test the importance of the neonatal parallels, we carried out a reverse analysis in order to identify the transcriptomic patterns distinguishing the MRL/MpJ adult mouse from the embryos, newborns and adults of the control strains. Among the transcripts down-regulated in the MRL/MpJ mouse relative to the adults, newborns, and embryos of the reference strain (Additional file 2), we found a remarkable overrepresentation of KRAB genes but none of those was associated with immune and wounding response. The transcripts up-regulated in the MRL/MpJ mouse, but not the reference embryos, newborns and adults (Additional file 2), were significantly enriched with the genes of PPAR signalling pathway. This set of PPAR signalling pathway genes included *Adipoq* and *Ucp1* but shared no genes with that which included the genes up-regulated in the adults of MRL/MpJ mouse and the newborns of the control strain (Table 7). This observation supports the possibility that the increased expression of *Adipoq* and *Ucp1* genes, the key markers of PPAR signalling, could be an exceptional trait of the MRL/MpJ mouse.

It is worth noting that the hearts of neonatal mice were found to regenerate after cardiac injury [72]. Our analysis reveals that the gene expression patterns in the heart of the adult MRL/MpJ mouse show a number of parallels with those of neonatal hearts. This finding entails a question as to whether these parallels are responsible for the healing response to myocardial injury reported in the MRL/MpJ mouse.

### Validation of the microarray results

**Fig. 5:**
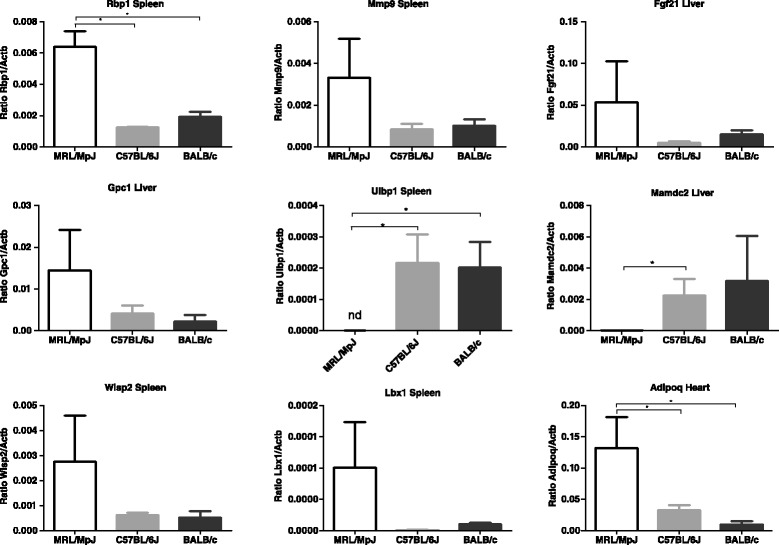
Quantitative real-time PCR validation of microarray results. The results represent mean and SD values calculated for three animals. The statistical significance has been determined using a two-tailed heteroscedastic Student’s *t*-test

We chose a selection of genes which show the differences in expression levels between the MRL/MpJ and the control strains in order to confirm the results of microarray with quantitative Real-Time PCR (Fig. 5). The genes were selected not only owing to differential expression but also due to their potential roles in wound repair and regeneration processes. E.g. Mmp9 has been reported to be responsible for basement membrane breakdown in the MRL/MpJ mouse, which is a key step in the regenerative healing observed in the strain [18] and *Lbx1* represents the homeobox genes. The *Mamdc2* gene, differentially expressed in four of the examined tissues of the MRL/MpJ mouse, encodes an extracellular region found in a functionally diverse set of proteins, mainly thought to have adhesive functions. Interestingly, MAMDC2 is a highly conserved protein which exhibits 100 % conservation in a number of mammals including mouse, human, chimpanzee, sheep, horse, dog, cat and the opossum *Monodelphis domestica*.

The results of qPCR analyses for ten selected transcripts in liver, spleen and heart are in agreement with our microarray results (Additional file 1: Table S2). It is worth noting that we have not detected the *Ulbp1* transcript in the spleen of the MRL/MpJ mouse (Fig. 5). *Ulbp1* is an immune response gene down-regulated in all examined tissue of the MRL/MpJ as compared to the control strains (Table 2). *Ulbp1* encodes a surface glycoprotein known to participate in the activation of signalling pathways in primary NK cells resulting in the production of chemokines and cytokines. As we are highlighting retinol pathways in the MRL/MpJ mouse as one of central findings of this study, we confirmed an enhanced expression of the *Rbp1* gene encoding retinol binding protein in the spleen of the MRL/MpJ mouse. The PCR quantitation was carried out for individual animals, unlike the microarray experiment, where the same samples were pooled. Consequently, the relatively high values of standard deviations correspond to individual variation among the examined animals.

## Conclusions

The global gene expression analysis revealed hundreds of transcripts differentially expressed in bone marrow, spleen, heart, liver and ear tissues of the MRL/MpJ mouse, known for high regenerative capacity, in comparison to two control strains the C57BL/6J and BALB/c, which do not display enhanced regeneration abilities. Twenty eight out of the transcripts were differentially expressed in the five examined tissues.

The down-regulated transcripts were greatly enriched with immune response genes in all examined tissues, most of them responsible for inflammatory response. The remarkable functional categories associated with the up-regulated transcripts are related to retinol metabolism, PPAR signalling, homeobox group, and keratin proteins and these are not shared by all examined tissues. The genes involved in wounding response and wound healing were enriched among both the up- and down-regulated transcripts, dependent on the tissue.

Gene ontology analyses showed an enrichment of retinol metabolism or retinoid binding pathway genes among those up-regulated in heart, spleen, ear and liver tissues of the MRL/MpJ mouse. The known role of retinoic acid during organism development and regeneration [36, 45–47] supports the concept that the distinguishing expression pattern of retinol metabolism genes is connected with the enhanced healing properties observed in the MRL/MpJ mouse.

In the hearts, spleens and bone marrow, a group of homeobox genes revealed a higher expression in the MRL/MpJ mouse in comparison to the control strains. Interestingly, we observed the up-regulation of the *Nanog* gene, the key marker of pluripotency in the adult heart of the healer strain. Among the transcripts up-regulated in the MRL/MpJ mouse, we found an enrichment of genes involved in wound healing response in the spleen and ear and in the PPAR signalling pathway in the heart.

We have also showed remarkable similarities between the expression patterns in the heart of adult MRL/MpJ mouse and those of the embryos and newborns of the reference strain. The key parallels included the up-regulation of several homeobox genes and the repression of a number of genes responsible for the positive regulation of immune response. Further, we identified several clusters of up-regulated genes shared by the MRL/MpJ adult mouse and the newborns, but not the embryos and adults of the control strains. These clusters correspond to PPAR signalling, retinol metabolism as well as a proteinase inhibitor genes, such as serpins. Neonatal mouse hearts have been shown to be able to regenerate up to day 5 after birth [72], so the transcriptomic similarities shared by the adult MRL/MpJ mouse with the newborns, but not the adults of the reference strain may be associated with enhanced regenerative and wound healing potential. What is more, the results indicate that the unusual features of the MRL/MpJ mouse may be related more to the neonatal than embryonic relics. Retention of foetal relics in adults has been investigated with regard to embryonic characteristics displayed by stem cells [24]. We provide a transcriptomic evidence of embryonic and neonatal parallels in the adult mouse. These parallels are not found in a subset of cells, but they have been deduced from the transcriptome analysis of tissues, thus reflecting the metabolic status of the whole system.

To our knowledge, this is the first transcriptomic study to show differentially expressed genes in a selection of tissues in the MRL/MpJ mouse by comparative analysis with two reference strains. The examination of gene expression changes in response to wounding and during healing in the ears and hearts of the MRL/MpJ mouse have been already reported [4, 10, 11, 26]. Our approach allowed us to make several novel observations, such as deducing the potential differences in PPAR signalling and retinol metabolism, as well as the neonatal transcriptomic parallels as the distinguishing features of the MRL/MpJ mouse. Further, we provided transcriptomic evidence on the differential regulation of homeobox and immune response genes in noninjured tissues. However, our particular attention was attracted by the differences in the expression profiles of genes associated with retinol metabolism because retinoids are small regulatory molecules potentially useful in pharmaceutical applications. We think that research on retinol signalling and retinoid profiles in the tissues of the MRL/MpJ mouse could provide clues how to enhance regenerative capacity in mammals.

## Methods

### Tissue samples and nucleic acid extraction

Tissue samples (hearts, livers, spleens, ears, blood and bone marrow) from 2-month-old females of the MRL/MpJ (stock #000486), C57BL/6J (stock #000664) strains were purchased from the Jackson Laboratory (Bar Harbor, USA) and those of 2-month-old females of the BALB/c strain were prepared in the Tri-City’s Academic Animal Experiment Centre of the Medical University of Gdańsk. The hearts, livers, spleens, and ears were collected from the same set of three individuals of each strain and bone marrow samples were collected from three additional sets including three animals of each strain. The hearts of the C57BL/6J mouse embryo and neonate females were collected in the Tri-City Academic Laboratory Animal Centre - Research and Services Centre, Medical University of Gdańsk. The ethical approvals for the collection of murine tissues no. 12/2013 and 18/2014 were issued by the Local Ethics Commission for Experimentation on Animals at the Medical University of Gdansk, Poland. All experiments were performed in accordance with relevant guidelines and regulations. The sex of murine embryos was determined by using PCR according to the method described by McFarlane *et al*. [73]. All tissues were collected in RNAlater stabilization reagent (Qiagen. cat. no. 76104), transported on dry ice and stored at −80 ° C. The solid tissues were disrupted in liquid nitrogen prior to RNA extraction. Total RNA was purified using the RNeasy Mini Kit (Qiagen. cat. no. 74104) coupled with on-column DNA digestion following the manufacturer’s protocols. The quality of total RNA was determined by Agilent 2100 Bioanalyzer (Agilent).

### RNA-Seq

Total RNA was extracted from single hearts of the MRL/MpJ, C57BL/6J and BALB/c mouse. Paired-end 300 bp sequencing was performed using HiSeq Illumina Sequencing Platforms (commercial service provided by the Genomics Core of Heflin Center for Genomic Science of University of Alabama, Birmingham, USA). RNA-Seq reads were processed with Galaxy platform (https://usegalaxy.org), including alignment using TopHat with mean inner distance of 150 bp, followed by CuffLink analysis carried out in order to determine differential genes expression between the murine strains under this study.

### Double stranded cDNA synthesis

Equal amounts of RNA templates extracted from three individuals from each murine strain were pooled for cDNA synthesis. First strand cDNA synthesis was performed with 400 units of Maxima Reverse Transcriptase (200 units/μl, ThermoScientificBio, cat. no. EP0742) using 3 μg of total RNA, 200 pmoles of oligo dT_15_, 8 μl of 5 x reaction buffer (250 mM Tris–HCl, 375 mM KCl, 15 mM MgCl_2_, 50 mM DTT) in a total volume of 40 μl. Second strand cDNA was synthesized using cDNA Synthesis System (Roche, cat. no. 11 117 831 001) according to the manufacturer’s protocol.

### Double stranded cDNA labelling and hybridization

Probe labelling and hybridization were performed with Roche NimbleGen kit using the standard protocol for eukaryotic RNA samples and NimbleGen mouse gene expression 12x135K array (Roche, cat. no. 05543797001). The cDNA samples were labelled with Cy3 using a NimbleGen One-Color DNA labelling kit (Roche, cat. no. 06370411001) and hybridized to slides using a NimbleGen hybridization system (Roche, cat. no. 05583683001). The slides were scanned using MS200 Scanner (Roche, NimbleGen) at 2 μm resolution by using high-sensitivity and autogain settings.

### Microarray data processing

The data from scanned images were processed and normalized using a robust multi-chip average (RMA) algorithm using DEVA 1.0.2 software with default settings (Roche) [74]. The results of microarray profiling were deposited in Gene Expression Omnibus Database under the accession number GSE64624 (http://www.ncbi.nlm.nih.gov/geo/query/acc.cgi?token=utmbmwkohdqvnwz&acc=GSE64624).

### Selection of differentially expressed transcripts

The ratios of linear expression values were calculated in order to select the transcripts showing at least a 2-fold difference in expression levels between the MRL/MpJ mouse strain and the control C57BL/6J and BALB/c strains. Calculations and data sorting were done in Excel spreadsheet.

### Gene set enrichment analysis

The gene set enrichment analyses were performed and functional annotations were assigned by using DAVID Bioinformatics Resource 6.7 9 (National Institute of Allergy and Infectious Diseases (NIAID) National Institute of Health) [28].

### Linear regression and scatter plots

Linear regression coefficients were determined and scatter plots were made using the DNASTAR Lasergene 12 software package (DNASTAR, Inc.; Madison, USA).

### Hierarchical clustering and statistics

Hierarchical clustering analysis (distance metric: Euclidean, linkage method: centroid (fast)) for differentially expressed transcripts was done by using the DNASTAR Lasergene 12 software package (DNASTAR, Inc.; Madison, USA). Hierarchical clustering for whole transcriptomic arrays and statistical tests was done using XLSTAT software package (Addinsoft).

### Quantitative real-time PCR

cDNA was synthesized using 200 ng of total RNA with 200 units of Maxima Reverse Transcriptase (ThermoScientific Bio. cat. no. EP0742), 100 pmoles of oligo dT_20_, and 4 μl of 5 x reaction buffer (250 mM Tris–HCl, 375 mM KCl, 15 mM MgCl_2_, 50 mM DTT) in a final volume of 20 μl. Approximately 5 ng of cDNA was used for subsequent real-time PCR reactions. The gene expression levels were calculated using the 2^-∆Ct^ method with the *Actb* as the reference gene. The results are presented as means ± SD. Real-time PCR reactions were carried out with FastStart Essential DNA Green Master mix (Roche, cat. no. 06402712001) on a LightCycler Nano® (Roche). The primers used in qRT PCR reaction are listed in Additional file 1: Table S3).

### Retinol blood concentrations

Retinol concentrations were determined in whole blood (0.3–0.5 ml) collected on EDTA from six females of 2-month-old mice of MRL/MpJ and C57BL/6J strains. Blood samples were mixed with 0.2 ml portions of saturated magnesium sulphate and sodium chloride, following double extraction with hexane: methylene chloride 3:2 (v/v). The organic phase was separated by centrifugation and evaporated. The dry residue was dissolved in 1 ml of methanol (HPLC grade) and filtered through a 0.22 micron syringe filter. Retinol concentrations were measured by reverse phase high-performance liquid chromatography using 600 pump system (Waters) and reversed phase HPLC column X-Terra RP18 5 μ, 150×4.6 mm (Waters) with UV-DAD 2996 detector set at a wavelength of 320 nm. The flow rate was 0.8 ml/min and the temperature was set at 23 °C. The analyses were done as a commercial service by the Laboratory of Wrocław Technology Park (Wrocław, Poland).

## Additional files

Additional file 1:
**Supplementary figures and tables.** Figure S1. Inter-strain comparisons of genome-wide gene expression profiles shown as scatter plots. The comparisons of genome-wide gene expression profiles between the MRL/MpJ mouse and the reference strains presented as scatter-plots. R2 - coefficient of linear regression. Figure S2. Hierarchical clustering of the genes showing up and down-regulation in the MRL/MpJ mouse. Hierarchical clustering of differentially expressed genes in ears, heart, liver, spleen and bone marrow tissues of the MRL/MpJ mouse versus the C57BL/6 J and BALB/c strains. Individual genes are clustered according to the dendrogram on the left and expression levels are represented in the heat map. Table S1. The genes differentially regulated in five examined tissues of the MRL/MpJ mouse. The genes showing at least a 2-fold difference in expression in five examined tissues in the MRL/MpJ mouse in comparison to the control C57BL/6 J and BALB/c strains. The results are presented as log_2_ of linear expression values. The genes for which the results were confirmed with RNA-seq in the heart are shown in red font. Table S2. The validation of microarray results by quantitative real-time PCR. * - *p*-value ≤ 0.05; Ht – heart; Liv – liver; Spl – spleen; ^+^ - no qPCR detection for *Ulbp1* in the MRL/MpJ spleen. Table S3. The list of PCR primers. (PDF 2276 kb) Additional file 2:
**The list of transcripts corresponding to the genes mentioned in the article.** The list of transcripts corresponding to the genes mentioned in the article and their expression levels is attached as an excel file. (XLSX 979 kb)
