# Supplementary material for: Transcriptome profiling reveals distinctive traits of retinol metabolism and neonatal parallels in the MRL/MpJ mouse
Source: BMC Genomics. 2015 Nov 14;16:926. doi: 10.1186/s12864-015-2075-2 (PMC4647819; doi:10.1186/s12864-015-2075-2)
Supplement: Additional file 1: — Supplementary figures and tables. Figure S1. Inter-strain comparisons of genome-wide gene expression profiles shown as scatter plots. The comparisons of genome-wide gene expression profiles between the MRL/MpJ mouse and the reference strains presented as scatter-plots. R2 - coefficient of linear regression. Figure S2. Hierarchical clustering of the genes showing up and down-regulation in the MRL/MpJ mouse. Hierarchical clustering of differentially expressed genes in ears, heart, liver, spleen and bone marrow tissues of the MRL/MpJ mouse versus the C57BL/6 J and BALB/c strains. Individual genes are clustered according to the dendrogram on the left and expression levels are represented in the heat map. Table S1. The genes differentially regulated in five examined tissues of the MRL/MpJ mouse. The genes showing at least a 2-fold difference in expression in five examined tissues in the MRL/MpJ mouse in comparison to the control C57BL/6 J and BALB/c strains. The results are presented as log2 of linear expression values. The genes for which the results were confirmed with RNA-seq in the heart are shown in red font. Table S2. The validation of microarray results by quantitative real-time PCR. * - p-value ≤ 0.05; Ht – heart; Liv – liver; Spl – spleen; + - no qPCR detection for Ulbp1 in the MRL/MpJ spleen. Table S3. The list of PCR primers. (PDF 2276 kb) [file 12864_2015_2075_MOESM1_ESM.pdf]

Figure S1. Inter-strain comparisons of genome-wide gene expression profiles shown as scatter plots.
The comparisons of genome-wide gene expression profiles between the MRL/MpJ mouse and the reference strains presented as scatter-plots. R2 - coefficient of linear regression

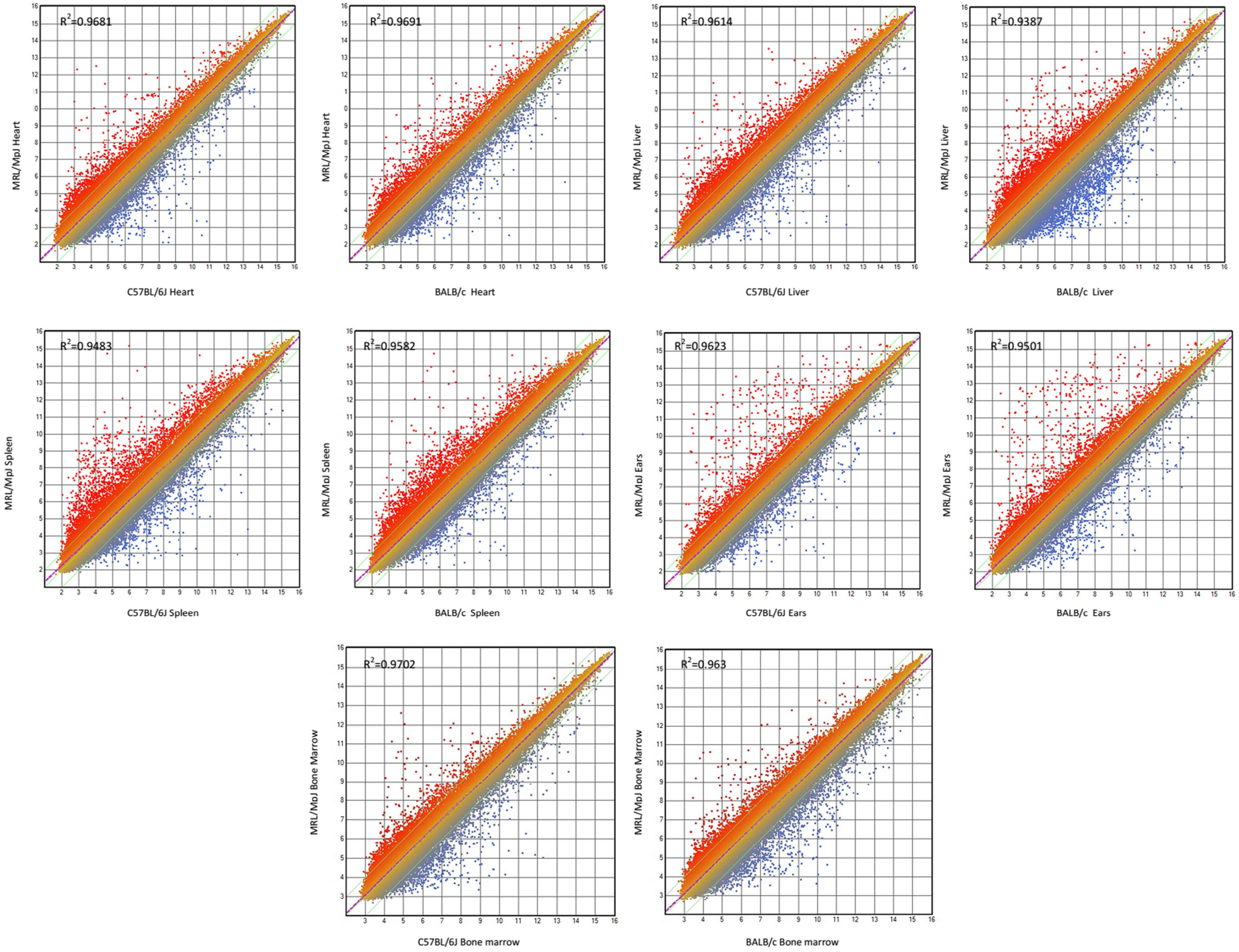

Figure S2. Hierarchical clustering of the genes showing up and down-regulation in the MRL/MpJ mouse.

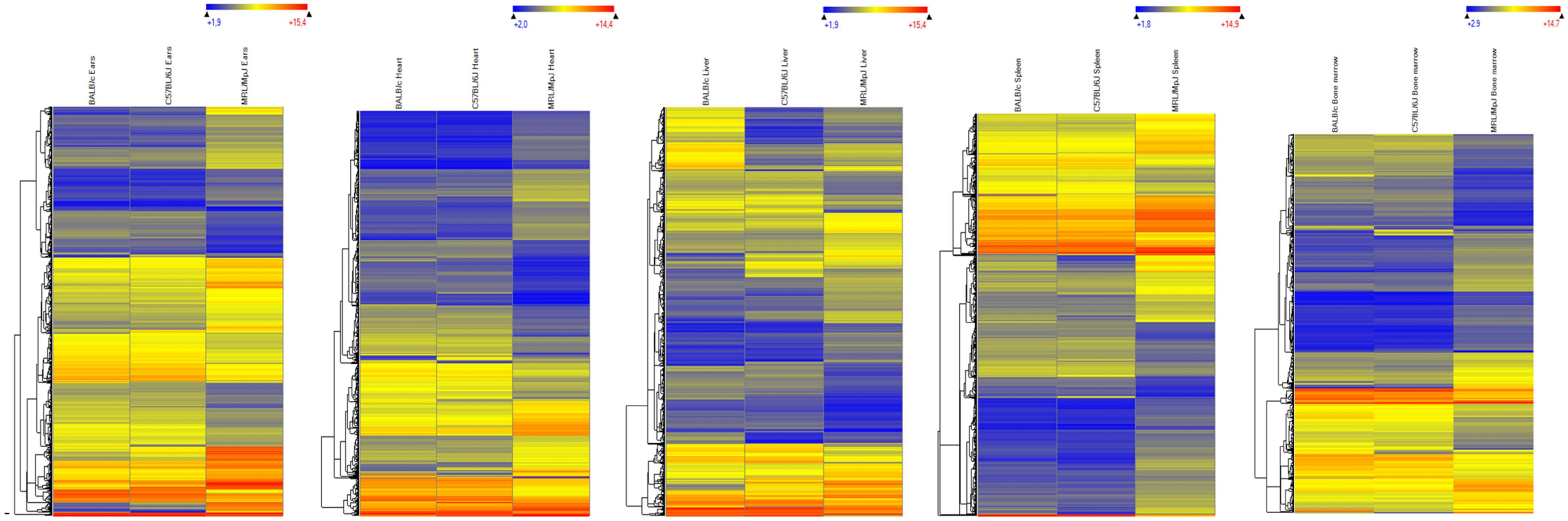

Table S1. The genes differentially regulated in five examined tissues of the MRL/MpJ mouse.

The genes showing at least a two-fold difference in expression in five examined tissues in the MRL/MpJ mouse in comparison to the control C57BL/6J and BALB/c strains. Results are presented as log_2 of linear expression values. The genes for which the results were confirmed with RNA-seq in the heart are shown in red font.

Gene/ transcript ID	BALB/c					C57BL6/J					MRL/MpJ					+1.00 … +16.00 Description
	Ear	Heart	Liver	Spleen	Bone marrow	Ear	Heart	Liver	Spleen	Bone marrow	Ear	Heart	Liver	Spleen	Bone Marrow	
**Up-regulated**																
AA465934	5.51	5.81	5.57	6.20	7.38	5.03	6.23	5.89	6.02	7.61	10.34	11.81	10.32	11.54	12.08	Mammary gland RCB-0527 Jyg-MC(B) cDNA
Akna	4.39	5.03	4.68	5.47	5.31	4.95	4.52	4.82	4.51	5.05	6.30	6.11	6.88	10.35	9.95	NOD-derived CD11c +ve dendritic cells cDNA; **AT-Hook Transcription Factor; Specifically activates the expression of the CD40 receptor and its ligand CD40L/CD154**
BC044745	5.43	6.37	6.15	7.65	7.05	5.47	5.42	5.23	5.58	5.06	10.30	9.87	9.94	12.08	12.06	cDNA clone IMAGE:5149239<br>LOC102638545 - long ncRNA of unknown function
Gm3556	7.04	6.49	7.08	8.19	7.73	7.19	5.68	7.50	8.43	7.63	8.96	7.94	8.84	9.58	8.88	Hypothetical protein LOC100041870
**Down-regulated**																
1110019D14Rik	6.59	8.24	8.07	6.68	6.86	6.39	8.23	8.18	6.92	7.15	3.85	2.79	5.57	5.06	5.04	18-day embryo whole body cDNA; ncRNA; **predicted as long intergenic non-coding RNA (Ensemble)**
1700029I01Rik	9.09	9.00	8.32	9.55	10.36	8.49	8.46	7.82	9.29	10.54	4.65	4.81	3.83	6.16	7.02	RIKEN cDNA 1700029I01 gene; ZNF41, pseudogene
1810058I24Rik	9.98	10.48	10.97	9.72	10.47	10.07	10.63	10.99	9.53	10.98	7.61	8.71	7.95	8.07	9.00	Adult inner ear cDNA, RIKEN full-length enriched library; **predicted as long intergenic non-coding RNA (Ensemble)**
4933439C10Rik	9.91	8.99	9.59	10.36	11.06	10.54	8.96	10.19	10.47	11.00	8.35	7.31	8.53	9.28	8.49	Adult male corpora quadrigemina cDNA; unclassified non-coding RNA gene (MGI)
5330426P16Rik XM_992800	8.55	9.49	6.13	9.35	10.01	7.86	9.22	6.94	8.87	9.43	5.49	6.95	4.44	5.93	6.27	RIKEN cDNA 5330426P16 gene (5330426P16Rik); **Long Intergenic Non-Protein Coding RNA 883**
5330426P16Rik AK151508	9.19	10.16	7.87	10.42	10.21	8.53	9.92	7.87	9.94	9.67	6.77	7.47	5.71	7.65	7.19	Bone marrow macrophage cDNA; **Long Intergenic Non-Protein Coding RNA 883**
9930104L06Rik	9.58	9.60	9.44	9.55	9.57	9.48	9.48	9.27	9.58	9.77	7.80	7.83	7.35	7.47	7.11	RIKEN cDNA 9930104L06 gene, mRNA (cDNA clone MGC:107426 IMAGE:30102988)
AK202070	8.16	7.73	5.52	8.07	7.52	8.59	7.73	6.90	8.37	7.57	5.27	5.58	4.47	5.99	6.11	cDNA, clone:Y1G0140E17
Akr1e1	9.43	10.24	11.88	9.06	8.73	9.85	10.93	12.12	9.06	9.10	6.96	8.96	9.39	6.11	6.71	Aldo-keto reductase family 1, member E1; **Catalyzes the NADPH-dependent reduction of 1,5-anhydro-D-fructose to**

																**1,5-anhydro-D-glucitol.**
BC022696	6.70	6.48	7.93	7.47	6.63	6.60	6.14	7.95	7.81	6.79	5.49	4.88	6.06	5.29	3.91	Clone IMAGE:4459415
C4b BC067409	12.91	11.13	15.01	12.42	11.82	12.91	10.54	14.80	12.49	11.18	11.15	9.46	13.67	10.73	9.55	Complement component 4B (Childo blood group); **A mediator of local inflammatory process.**
C4b BC039141	12.96	11.25	14.82	12.82	10.98	12.96	10.70	14.70	12.75	10.48	11.86	9.65	13.65	11.37	9.33	Complement component 4B (Childo blood group); **A mediator of local inflammatory process.**
Ceacam1	8.73	10.77	13.82	11.09	13.25	7.94	11.10	13.78	11.37	12.72	5.98	9.75	12.33	8.64	10.98	carcinoembryonic antigen-related cell adhesion molecule 1; **May play a role in angiogenesis, tumor suppression, metastasis and the modulation of innate and adaptive immune responses.**
Cradd	8.56	9.07	9.72	7.08	6.82	8.66	9.50	10.24	7.45	7.13	5.49	6.68	7.58	3.87	5.22	CASP2 and RIPK1 domain containing adaptor with death domain; **Apoptotic adaptor molecule specific for caspase-2 and FASL/TNF receptor-interacting protein RIP.**
Dusp12	7.68	6.53	8.77	6.82	7.10	7.72	6.27	8.25	7.27	7.23	4.84	4.04	4.26	5.24	5.44	Dual specificity phosphatase 12; **Serine/Threonine Specific Protein Phosphatase.**
Fam173b	6.33	7.41	6.16	6.11	7.67	5.89	7.65	6.67	5.95	7.74	4.60	5.75	4.39	4.38	5.89	Family with sequence similarity 173, member B
Gm10060	8.67	8.05	8.41	8.94	10.11	8.35	7.72	8.38	8.21	9.82	7.26	6.22	7.14	7.18	8.70	Similar to p47 protein (LOC100041567); **NSFL1 (p97) cofactor (p47) pseudogene**
LOC625360	4.37	5.24	4.89	10.12	9.08	4.33	5.73	5.03	9.94	9.04	3.05	3.02	3.37	6.40	5.31	Similar to 2-cell-stage, variable group, member 3
Nsun3	10.13	9.76	10.63	9.51	9.75	10.10	9.97	10.84	9.63	9.98	7.72	8.44	9.24	7.52	7.93	NOL1/NOP2/Sun domain family member 3; **May have S-adenosyl-L-methionine-dependent methyl-transferase activity.**
Slc25a17	11.19	9.60	10.92	9.64	10.07	11.25	9.74	11.56	9.64	10.35	8.41	7.98	8.15	5.71	8.50	Solute carrier family 25 (mitochondrial carrier, peroxisomal membrane protein), member 17; **May catalyze the transport of free CoA, FAD and NAD(+) from the cytosol into the peroxisomal matrix by a counter-exchange mechanism.**
Stard6	6.12	7.69	5.53	7.65	7.28	6.36	7.59	5.38	7.17	7.16	3.25	4.11	3.69	4.15	4.37	StAR-related lipid transfer (START) domain containing 6; **May be involved in the intracellular transport of sterols and other lipids.**
Tmem18	10.07	9.73	10.66	9.98	10.14	9.77	9.60	10.10	9.14	9.13	5.20	5.54	5.01	2.85	5.60	NOD-derived CD11c +ve dendritic cells cDNA; **Transmembrane Protein 18; Transcription repressor**
Ulbp1	7.22	8.29	4.69	9.24	10.79	6.66	8.18	6.29	9.18	10.63	5.03	6.86	2.80	5.49	7.64	UL16 binding protein 1; **ULBPs activate multiple signaling pathways in primary NK cells, resulting in the production of cytokines and chemokines**
Zfp870	6.91	7.98	7.18	7.02	6.47	6.45	8.22	7.03	6.94	6.26	4.03	6.33	5.79	5.77	4.06	zinc finger protein 870

Table S2. The validation of microarray results by quantitative real-time PCR.

Transcript ID	Official Gene Symbol	Tissue	Fold ratio (microarray)		Fold ratio (qPCR)		Fold Ratio (RNAseq)	
			MRL/MpJ - C57BL/6J	MRL/MpJ - BALB/c	MRL/MpJ-C57BL/6J	MRL/MpJ-BALB/c	MRL/MpJ-C57BL/6J	MRL/MpJ-BALB/c
BC012701	*Ucp1*	Ht	4.41	14.19	15.21	211.71	33.59	∞
BC028770	*Adipoq*	Ht	2.78	4.13	4.06*	13.79*	5.58	13.74
BC091751	*Rbp1*	Spl	4.81	3.16	5.17*	3.33*	-	-
BC046991	*Mmp9*	Spl	3.48	2.68	3.98	3.25	-	-
BC049592	*Fgf21*	Liv	4.39	2.00	10.85)	3.63	-	-
BC042773	*Mamdc2*	Liv	0.022	0.037	4.31E-03	3.05E-03	-	-
BC062902	*Gpc1*	Liv	3.63	2.96	3.54	6.79	-	-
NM_029975	*Ulbp1*	Spl	0.08	0.07	0.00E+00^{+}*	0.00E+00^{+}*	-	-
BC119177	*Lbx1*	Spl	2.35	2.77	108.03	9.61	-	-
BC032877	*Wisp2*	Spl	2.86	3.70	4.43	5.32	-	-

* - p-value < 0.05;

Ht – heart; Liv – liver; Spl – spleen;

$^{+}$ - no qPCR detection for *Ulbp1* in the MRL/MpJ spleen.

Table S3. The list of PCR primers.

Transcript ID (NCBI)	Gene name	Amplicon length (bp)	Primer sequence
BC028770	*Adipoq*	152	F: TCCTGGAGAGAAGGGAGAGAAA
			R: GATACACATAAGCGGCTTCTCC
BC012701	*Ucp1*	195	F: TCCAAGAGTACTTCTCTTCAGG
			R: TAACTCTGTAAGCATTGTAGGTCC
BC046991	*Mmp9*	128	F: TAAAGACGACATAGACGGCATC
			R: GAGGTATAGTGGGACACATAGT
BC091751	*Rbp1*	136	F: TCGGAACTATATCATGGACTTCCAA
			R: CTCCTTCTCTCCCTTCTGCACA
BC049592	*Fgf21*	148	F: TCATTCAAATCCTGGGTGTCAAA
			R: GCTTCAGACTGGTACACATTGTAA
BC042773	*Mamdc2*	151	F: TTTGATAGAAGGTGTCCTGGGA
			R: GAACCAGTTCACATTGGGATTC
BC062902	*Gpc1*	123	F: ATCTACGGGGCTAAGGGCTTTA
			R: GGCCAAATTCTCCTCCATCTCA
NM_029975	*Ulbp1*	168	F: TAAAGAAGACTACAGAGGGCGA
			R: GTCTTAATTTACAAGAGACTCCGAA
BC032877	*Wisp2*	121	F: CCCAGGAGAATACAGGTGCCAG
			R: GAGTGACAAGGGCAGAAAGTTG
BC119177	*Lbx1*	142	F: CAAGACCTTTAAGGGGCTGGAG
			R: CAACTCGTAGATCTGGTGGTTG
NM_007393.3	*Actb*	500	F: TCAGAAGGACTCCTATGTGG
			R: TCTCTTTGATGTCACGCACG
